# Risk Factors for Chronic Kidney Disease in Older Adults with Hyperlipidemia and/or Cardiovascular Diseases in Taipei City, Taiwan: A Community-Based Cross-Sectional Analysis

**DOI:** 10.3390/ijerph17238763

**Published:** 2020-11-25

**Authors:** Horng-Jinh Chang, Kuan-Reng Lin, Junn-Liang Chang, Meng-Te Lin

**Affiliations:** 1 Department of Management Sciences, Tamkang University, New Taipei 25137, Taiwan; chj@mail.tku.edu.tw; 2 Department of Pathology & Laboratory Medicine, Taoyuan Armed Forces General Hospital, Taoyuan 32551, Taiwan; junn9liang@yahoo.com.tw; 3 Biomedical Engineering Department, Ming Chuan University, Taoyuan 333, Taiwan

**Keywords:** chronic kidney disease, glomerular filtration rate, hyperlipidemia, cardiovascular disease, interaction

## Abstract

This cross-sectional study aimed to compare risk factors for chronic kidney disease (CKD) in older adults with or without dyslipidemia and/or cardiovascular diseases (CVD) in Taipei City, Taiwan. The data on 2912 participants with hyperlipidemia and/or CVD and 14,002 healthy control participants derived from the Taipei City Elderly Health Examination Database (2010 to 2011) were analyzed. The associations between conventional CKD risk factors and CKD were comparable between participants with and without hyperlipidemia. Participants with high uric acid and BUN had a higher risk of CKD if they also had hyperlipidemia and CVD [odds ratio (OR) in uric acid = 1.572, 95% CI 1.186–2.120, *p* < 0.05; OR in BUN = 1.271, 95% CI 1.181–1.379, *p* < 0.05]. The effect was smaller in participants with hyperlipidemia only (OR in uric acid = 1.291, 95% CI 1.110–1.507, *p* < 0.05; OR in BUN = 1.169, 95% CI 1.122–1.221, *p* < 0.05). The association between uric acid/BUN and CKD was also observed in the healthy population and participants with CVD only. In conclusion, older adults with hyperlipidemia and CVD are at high of CKD. Physicians should be alert to the potential for CKD in older patients with hyperlipidemia and CVD.

## 1. Introduction

Chronic kidney disease (CKD) is a worldwide health problem with a steady annual increase in occurrence of approximately 6%, and significant differences in prevalence between populations [1,2]. CKD represents an important public health issue because such patients have an increased risk of end-stage renal disease (ESRD). Taiwan has a high prevalence of both CKD [3] and ESRD [4]. Significantly elevated cardiovascular morbidity and mortality have been observed in the course of CKD. In patients with CKD, the cardiovascular mortality rate is 10 to 20 times higher than in the general population, and in the ESRD population, it is 20–30 times higher [5,6]. The spectrum of cardiovascular diseases (CVD) in the CKD population includes arterial vascular disease such as atherosclerosis and arteriosclerosis, concentric left ventricular hypertrophy, heart failure, and non-atherosclerotic CVD, which becomes dominant in more advanced stages of CKD [7,8]. However, unravelling the exact mechanisms and causal pathways linking CKD and CVD remains a challenge. 

Research indicates that the increased cardiovascular risk in patients with CKD is multifactorial and involves both conventional (hypertension, diabetes, dyslipidemia) and kidney-specific risk factors, such as enhanced activity of the renin–angiotensin system, sympathetic overactivity, endothelial dysfunction (related to the accumulation of asymmetric dimethylarginine, chronic inflammatory state, and oxidative stress), hyperphosphatemia, and CKD-associated metabolic bone disorder [8,9,10]. Disturbances in the lipid profile, an important factor associated with the development of CVD, may lead to dyslipidemia and the accumulation of atherogenic particles [11]. Hypertriglyceridemia, higher levels of low-density lipoprotein cholesterol, lipoprotein(a) particles, and apolipoprotein B (Apo B)-containing lipoproteins, as well as low high-density lipoprotein (HDL) levels, are the most frequent alterations observed in CKD patients. CKD not only fuels the reduction in HDL levels, but it also modifies the composition of this lipoprotein, for example, by diminishing plasma levels of the main HDL components, namely apoA-I and apoA-II [12,13]. 

The benefits of screening high-risk populations and estimating the progression of CKD are well established [14]. Planning a screening/prevention strategy for a specific population with CVD or dyslipidemia is a major public health challenge. Due to the higher incidence of CKD in patients with dyslipidemia and CVD in several European countries [15,16,17,18], it is reasonable to assume that patients with dyslipidemia and/or CVD are at a higher risk for developing CKD. Furthermore, due to population aging, the number of people age 65 years and over worldwide is estimated to be about 1.6 billion by 2050, resulting in elevated medical expenditures and social burden [19]. Hence, this retrospective study aimed to investigate the extent to which the conventional CKD risk factors are associated with the risk for developing CKD in older adults aged 65 and older with or without dyslipidemia and/or CVD, using a health examination database of a community-dwelling elderly population in Taipei City, Taiwan. The anticipated results will give a representative overview of the impact of dyslipidemia and CVD on CKD in East Asia and may provide insights into the development of effective CKD prevention strategies for older patients with dyslipidemia and/or CVD.

## 2. Materials and Methods

### 2.1. Population and Definition

The present study utilized data derived from the Taipei City Elderly Health Examination Database (2010 to 2011), which collected health examination data from community-dwelling Taipei citizens aged 65 years or older. Taipei City, located in northern Taiwan, is the capital of Taiwan. The Taipei City Elderly Health Examination Database sponsored by the Department of Health, Taipei City Government, has been used for research as previously described [20,21,22,23]. The protocol of this study was reviewed and approved by the Institutional Review Board (IRB) of Taipei City Hospital (TCHIRB-10514118-W). In this cross-sectional study, participants who had missing values for age, sex, or serum creatinine level were excluded because all these variables were required to calculate the estimated Glomerular Filtration Rate (eGFR) based on the Chronic Kidney Disease Epidemiology Collaboration formula [24]. In addition, 91 participants were excluded because of cancer, nephrectomy, kidney transplantation, or ESRD and waiting for a renal transplant. Finally, a total of 16,914 (7533 male and 9381 female) older participants (mean age 74.9 years) were included in the study.

### 2.2. Data Extraction

The physical examination data and physical and mental questionnaire data included sex, age, body mass index (BMI), clinical laboratory data, and other parameters, which were defined as descripted in previous studies [20,21,22,23]. The lifestyle behaviors include smoking, exercise habit, alcohol drinking, and betel nut chewing. The behavioral data was based on a standardized self-administered questionnaire developed by the Health Promotion Administration.

The blood samples were analyzed at the hospital laboratory, and serum creatinine was measured using the direct method. CKD was defined as estimated glomerular filtration rate (eGFR) < 60 mL/min/1.73 m^2^ [25]. Urine albumin-to-creatinine ratio (UACR) measurement was not available in this study. eGFR can be calculated for specific ethnic groups by formulas based on the serum creatinine concentration, age, and sex, using the equation of the Chronic Disease Epidemiology Collaboration, as follows [26]:

Male: eGFR (mL/min/1.73 m^2^) = 186 × serum creatinine (mg/dL)^−1.154^ × age (years)^−0.203^ (1)

Female: eGFR (mL/min/1.73 m^2^) = 186 × serum creatinine (mg/dL)^−1.154^ × age (years)^−0.203^ × 0.742. (2)

In addition, proteinuria was determined by dipstick test as previously described [27], and the presence (positive: 1+ or more) or absence of proteinuria was recorded and analyzed in this study. The presence of hyperlipidemia, CVD, and other comorbidities was indicated based on the self-reported medical condition and medication use.

### 2.3. Statistical Analysis

Characteristics between individuals with low and high eGFR were compared; continuous variables were presented as mean and standard deviation, tested using the t test; categorical variables were presented as count and percentage, tested using the chi-square test. The association between risk factors and the eGFR level (low eGFR: eGFR < 60 mL/min/1.73 m^2^ and high eGFR: eGFR ≥ 60 mL/min/1.73 m^2^) was assessed by logistic regression. Multivariate logistic regression was performed to adjust for variables that were significant in the univariate regression model. Stratified analyses were performed based on dyslipidemia and CVD to explore the association between risk factors and the eGFR level. The significance level was set as two-sided *p* < 0.05. All statistical analyses were performed using the statistical software R, version 3.6.1 (R Foundation for Statistical Computing, Vienna, Austria).

## 3. Results

### 3.1. Clinical Characteristics of the Study Population

A total of 16,914 older participants were included in this study, with a mean age of 74.86 years. The patients’ characteristics are presented in Table 1.

This study cohort was slightly female-predominant (55.46%) with the prevalence of comorbidities as follows: hypertension, 8.14%; diabetes mellitus, 2.93%; hyperuricemia/gout, 7.17%; proteinuria, 16.66%; and CVD, 9.31%. Moreover, the majority of participants were married/cohabiting (74.07%) and 48.73% had normal BMI. In terms of socioeconomic characteristics, 56.8% had a high school diploma or higher and 97.66% were financially better than poor. Most participants had a regular exercise habit (38.28%), did not drink (81.61%), had no current smoking habit (95.24%), and reported no betel nut chewing (99.35%).

### 3.2. The Association of Laboratory Values, Comorbidity, and Future Risk of CKD 

**Figure 1:**
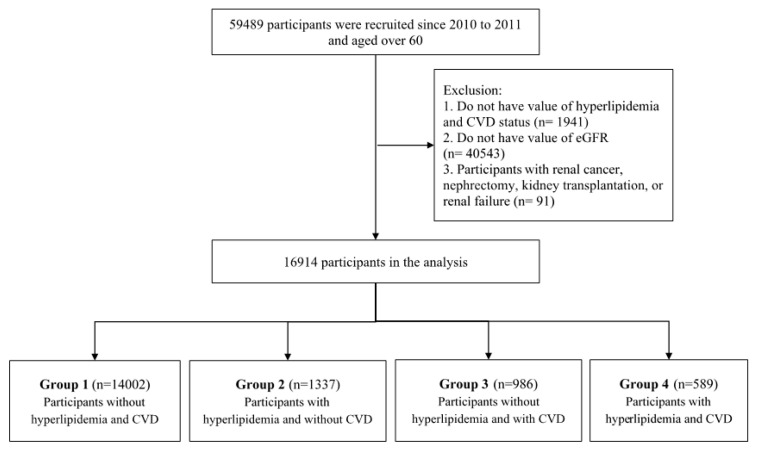
Flow chart of study population.

Participants were divided into four groups based on dyslipidemia and CVD status: Group 1: participants without dyslipidemia and CVD; Group 2: participants with dyslipidemia only; Group 3: participants with CVD only; and Group 4: participants with dyslipidemia and CVD (Figure 1).

Demographic, socioeconomic, lifestyle factors, laboratory data, and comorbidities of the four groups, stratified by eGFR level (eGFR < 60: CKD; eGFR ≥ 60: non-CKD), are shown in Table 2.

Aging, high uric acid, high blood urea nitrogen (BUN), proteinuria, and a history of hyperuricemia/gout were significantly associated with CKD in all four groups (all *p* < 0.05, Table 2). Male sex, obesity, high triglyceride, HDL-C, and prior hypertension were significantly associated with CKD in Group 1, Group 2, and Group 3; however, low income, low education, and high exercise habit were significantly associated with CKD in Group 1 (all *p* < 0.05, Table 2).

Univariate logistic regression analysis found a similar relationship between these factors (having a significant difference between CKD and non-CKD individuals in each Group in Table 2) and the risk of CKD in these subgroups (all *p* < 0.05, Table 3). In multivariate logistic regression analysis adjusted for the risk factors associated with CKD as shown in Table 3, we found similar relationships between these factors (having a significant difference between CKD and non-CKD individuals in each Group in Table 2) and the risk of CKD in these subgroups (all *p* < 0.05, Table 4). However, the effect of low income on the risk of CKD did not differ significantly by subgroup after adjustment for conventional risk factors (*p* > 0.05, Table 4).

## 4. Discussion

In the present study, male sex, greater age, high triglyceride, high uric acid, high BUN, high proteinuria, and prior hyperuricemia/gout were risk factors for CKD in participants without hyperlipidemia or CVD. We also found that BMI, marital status, income level, smoking, alcohol intake, betel nut chewing, fasting glucose, triglyceride, and history of diabetes or cancer were not associated with CKD in participants without hyperlipidemia or CVD. Consistently, several studies indicated that male sex and physical inactivity are two conventional risk factors for CKD in adults [28,29,30]. In our study, participants were divided into four groups, depending on the presence of dyslipidemia and CVD, and significant associations between these conventional risk factors and CKD were observed in certain groups, but not in all four groups.

Low income is a critical predictive factor for CKD. Consistently, our data also indicated that low income was significantly associated with a high risk of CKD in participants without hyperlipidemia or CVD. On the other hand, people with lower socioeconomic status are often associated with many common health risk factors including smoking [31], second-hand tobacco smoke exposure [32], alcohol consumption [33], unhealthy diets [34], and betel nut chewing [35]. Cigarette smoking and betel nut chewing have been reported to be associated with CKD [36,37]; however, no significate associations between these common health risk factors and CKD in all four groups were observed in the present study. The discrepancy between the current findings and the results of two abovementioned studies might be partially due to the differences in age range, inclusion criterion, participant grouping, and/or adjustment for socioeconomic status [36,37]. Hence, further large-scale investigation is warranted to clarify the association between individual socioeconomic status-related factors and CKD in older adults with hyperlipidemia and/or CVD.

Older participants with high uric acid and BUN had a higher risk of CKD than those without it in all four groups, and the association was very high after adjustment for conventional risk factors in the present study. The prevalence of hyperuricemia appears higher in older Taiwanese and the total population [38,39], which may, in part, contribute to the high incidence of ESRD observed in Taiwan [40]. Similarly, an elevated prevalence of hyperuricemia in patients with CKD and nonalcoholic fatty liver disease (NAFLD) in Iran was recently reported [41]. Several studies revealed that hyperuricemia is significantly associated with CKD in the general population [29,39]; however, the present study further found that older adults with hyperuricemia are at a much higher risk of developing CKD compared to the general population. Taken together, we suggest that hyperuricemia and BUN, compared to other conventional risk factors, might be associated with a greater risk of developing CKD and ESRD.

It has been documented that awareness of CKD in people who displayed markers and/or clinical manifestations associated with CKD, such as increasing age, obesity, CVD, and hyperlipidemia, is extremely low [42,43]. Consequently, it is a high priority for health professionals to promote awareness of CKD in high-risk populations, thereby facilitating early detection of CKD [43]. The present study found that uric acid and BUN levels were strongly associated with CKD in older patients with or without hyperlipidemia and CVD, suggesting the potential of uric acid and BUN levels as biomarkers for CKD in older adults aged 65 and over. Notably, hyperlipidemia is a risk factor for hyperuricemia in patients with CKD and NAFLD [41]. Furthermore, dyslipidemia and CVD have been demonstrated to be independently associated with CKD in the general population [44,45]. Taken together, older patients with hyperlipidemia and/or CVD are at very high risk of developing CKD, and their awareness of CKD should be enhanced by health professionals.

In the present study, the harmful effects of high lipid levels (triglyceride, total cholesterol, and HDL) on developing CKD were only observed in the general population and participants with hyperlipidemia only, but not in those with CVD only. Male participants were at a higher risk of CKD than their female counterparts; however, the negative impact of high lipid levels on CKD was less severe in males compared to that of females [29]. Obesity was a risk factor related to high lipid levels. In the present study, obesity and high lipid levels produced comparable harmful effects on CKD, suggesting the importance of hyperlipidemia and obesity in the prediction of CKD. Renal lipid accumulation was demonstrated to be nephrotoxic and may be involved in the progression of CKD [46]. However, further research is warranted to disclose the underlying mechanism responsible for all associations with CKD observed in older adults with hyperlipidemia and/or CVD.

The strength of this study is the utilization of the Taipei City Elderly Health Examination Database, which allows for comprehensive investigation of health issues in older adults aged 65 and over. However, several limitations of this study have to be discussed. First, no cause–effect relationships could be explored and established in this cross-sectional study. Second, self-reported questionnaires were used to collect data on lifestyle factors, so the possibility of recall bias cannot be ruled out. Finally, quantitative data on proteinuria level was not available in this study. Furthermore, the current findings collected from northern Taiwan should be confirmed by additional large-scale cross-sectional and longitudinal studies conducted in different geographic areas.

## 5. Conclusions

Sex, age, income, lipid levels, proteinuria, and prior hyperuricemia/gout represent potential candidates for developing an effective screening/prevention strategy for CKD in older adults with or without hyperlipidemia. However, smoking, alcohol intake, and betel nut chewing were not good candidates for CKD prevention in these populations. In addition, high uric acid or BUN levels may also be considered in the screening strategy for CKD in older patients with hyperlipidemia and CVD. While implementing a specific CKD screening/prevention strategy for older patients with hyperlipidemia and/or CVD, the awareness of CKD in such high risk populations should be enhanced by health professionals.

## Figures and Tables

**Table 1:** Overall characteristics of the total study population.

Variables	Total ^a^ (*n* = 16,914)
**Demographics**	
Age, years	74.9 ± 6.74
Gender	
Female	9381 (55.46%)
Male	7533 (44.54%)
Marital status	
Married/cohabiting	12,528 (74.07%)
Widowed/divorced/separated	3704 (21.89%)
Never married	664 (3.93%)
Missing value	18 (0.11%)
BMI ^b^ level, kg/m^2^	
Underweight	754 (4.46%)
Normal	8242 (48.73%)
Overweight	5033 (29.76%)
Obese	2741 (16.20%)
Missing value	144 (0.85%)
**Socioeconomics**	
Educational attainment	
With high school diploma or higher degree	9607 (56.80%)
Without high school diploma	7307 (43.20%)
Income level	
Not poor	16,519 (97.66%)
Poor	395 (2.34%)
**Lifestyle**	
Exercise habit	
No	2522 (14.91%)
Occasional	5694 (33.67%)
Regular	6475 (38.28%)
Missing value	2223 (13.14%)
Alcohol drinking	
No	13,804 (81.61%)
Yes	3088 (18.26%)
Missing value	22 (0.13%)
Current smoking	
No	16,109 (95.24%)
Yes	784 (4.64%)
Missing value	21 (0.12%)
Betel nut chewing	
No	16,804 (99.35%)
Yes	65 (0.38%)
Missing value	45 (0.27%)
**Comorbidity ^c^**	
Hypertension	1377 (8.14%)
Diabetes	496 (2.93%)
Hyperlipidemia	1926 (11.39%)
Hyperuricemia/gout	1213 (7.17%)
Urinary tract stones	47 (0.28%)
CVD	1575 (9.31%)
Cancer	4029 (23.82%)
**Laboratory examinations**	
Fasting glucose, mg/dL	101.4 ± 20.69
Total cholesterol, mg/dL	190.1 ± 33.70
Triglyceride, mg/dL	115.7 ± 69.23
HDL, mg/dL	55.1 ± 15.19
Uric acid, mg/dL	5.7 ± 1.57
BUN, mg/dL	17.2 ± 6.11
Creatinine, mg/dL	0.9 ± 0.75
Triglyceride/HDL, ratio	2.4 ± 2.25
ratio < 3.29	13,374 (79.07%)
ratio ≥ 3.29	3411 (20.17%)
Proteinuria	2818 (16.66%)

^a^ Data on the total study population were presented as mean ± standard deviation or number of participants (% of total cohort); ^b^ Underweight: BMI < 18.5; Normal: 8.5 ≤ BMI < 24; Overweight: 24 ≤ BMI < 27; Obese: BMI ≥ 27; ^c^ Some participants had multiple comorbidities; Abbreviation: BMI, body mass index; CVD, cardiovascular disease; HDL, high density lipoprotein; BUN, blood urea nitrogen.

**Table 2:** Characteristics of subjects with low and high estimated Glomerular Filtration Rate (eGFR), stratified by dyslipidemia and cardiovascular disease status.

	Group 1			Group 2			Group 3			Group 4		
	Low eGFR	High eGFR	*p*-Value	Low eGFR	High eGFR	*p*-Value	Low eGFR	High eGFR	*p*-Value	Low eGFR	High eGFR	*p*-Value
	(*n* = 3450)	(*n* = 10,552)		(*n* = 231)	(*n* = 1106)		(*n* = 215)	(*n* = 771)		(*n* = 120)	(*n* = 469)	
**Demographics**												
Age, years	77.9 ± 7.04	74.1 ± 6.52	**<0.0001** ^a^	75.1 ± 6.00	72.7 ± 5.20	**<0.0001** ^a^	77.9 ± 6.71	75.1 ± 6.76	**<0.0001** ^a^	76.9 ± 6.54	73.2 ± 5.37	**<0.0001** ^a^
Gender (%)			**<0.0001** ^b^			**0.04** ^b^			0.77 ^b^			0.15 ^b^
Female	1731 (50.17%)	5965 (56.53%)		139 (60.17%)	745 (67.36%)		99 (46.05%)	366 (47.47%)		61 (50.83%)	275 (58.64%)	
Male	1719 (49.83%)	4587 (43.47%)		92 (39.83%)	361 (32.64%)		116 (53.95%)	405 (52.53%)		59 (49.17%)	194 (41.36%)	
Marital status (%)			**<0.0001** ^b^			0.34 ^b^			**0.04** ^b^			0.40 ^b^
Married/cohabiting	2401 (69.59%)	7861 (74.50%)		192 (83.12%)	882 (79.75%)		145 (67.44%)	583 (75.62%)		90 (75.00%)	374 (79.75%)	
Widowed/divorced/separated	888 (25.74%)	2239 (21.22%)		37 (16.02%)	200 (18.08%)		62 (28.84%)	161 (20.88%)		29 (24.17%)	88 (18.76%)	
Never married	156 (4.52%)	440 (4.17%)		2 (0.86%)	24 (2.17%)		8 (3.72%)	26 (3.37%)		1 (0.83%)	7 (1.49%)	
Missing value	5 (0.15%)	12 (0.11%)		0 (0%)	0 (0%)		0 (0%)	1 (0.13%)		0 (0%)	0 (0%)	
BMI ^c^ level (%)			**<0.0001** ^b^			**0.02** ^b^			**0.001** ^b^			0.47 ^b^
Underweight	154 (4.46%)	496 (4.70%)		6 (2.60%)	43 (3.89%)		12 (5.58%)	31 (4.02%)		2 (1.67%)	10 (2.13%)	
Normal	1518 (44.00%)	5324 (50.45%)		95 (41.13%)	547 (49.46%)		85 (39.53%)	401 (52.01%)		48 (40.00%)	224 (47.76%)	
Overweight	1056 (30.61%)	3063 (29.03%)		81 (35.06%)	351 (31.73%)		69 (32.09%)	231 (29.96%)		42 (35.00%)	140 (29.85%)	
Obese	686 (19.89%)	1574 (14.92%)		49 (21.21%)	162 (14.65%)		48 (22.33%)	102 (13.23%)		27 (22.50%)	93 (19.83%)	
Missing value	36 (1.04%)	95 (0.90%)		0 (0%)	3 (0.27%)		1 (0.47%)	6 (0.78%)		1 (0.83%)	2 (0.43%)	
**Socioeconomics**												
Educational attainment (%)			**<0.0001** ^b^			0.08 ^b^			0.64 ^b^			0.38 ^b^
With high school diploma or higher degree	1671 (48.43%)	5870 (55.63%)		152 (65.80%)	794 (71.79%)		149 (69.30%)	519 (67.32%)		88 (73.33%)	364 (77.61%)	
Without high school diploma	1779 (51.57%)	4682 (44.37%)		79 (34.20%)	312 (28.21%)		66 (30.70%)	252 (32.68%)		32 (26.67%)	105 (22.39%)	
Income level (%)			**<0.0001** ^b^			0.14 ^b^			0.80 ^b^			0.50 ^b^
Not poor	3313 (96.03%)	10,324 (97.84%)		229 (99.13%)	1104 (99.82%)		209 (97.21%)	754 (97.80%)		119 (99.17%)	467 (99.57%)	
Poor	137 (3.97%)	228 (2.16%)		2 (0.87%)	2 (0.18%)		6 (2.79%)	17 (2.20%)		1 (0.83%)	2 (0.43%)	
**Lifestyle**												
Exercise habit (%)			0.09 ^b^			0.85 ^b^			0.54 ^b^			0.05 ^b^
No	447 (12.96%)	1656 (15.69%)		26 (11.25%)	172 (15.55%)		24 (11.16%)	113 (14.66%)		15 (12.50%)	69 (14.71%)	
Occasional	1088 (31.54%)	3756 (35.60%)		57 (24.68%)	330 (29.84%)		62 (28.84%)	234 (30.35%)		37 (30.83%)	130 (27.72%)	
Regular	1111 (32.20%)	4263 (40.40%)		70 (30.30%)	438 (39.60%)		69 (32.09%)	317 (41.11%)		26 (21.67%)	181 (38.59%)	
Missing value	804 (23.30%)	877 (8.31%)		78 (33.77%)	166 (15.01%)		60 (27.91%)	107 (13.88%)		42 (35.00%)	89 (18.98%)	
Alcohol drinking (%)			0.32 ^b^			0.78 ^b^			0.48 ^b^			0.18 ^b^
No	2813 (81.54%)	8682 (82.28%)		187 (80.95%)	882 (79.75%)		164 (76.28%)	607 (78.73%)		90 (75.00%)	379 (80.81%)	
Yes	633 (18.35%)	1856 (17.59%)		44 (19.05%)	222 (20.07%)		51 (23.72%)	163 (21.14%)		30 (25.00%)	89 (18.98%)	
Missing value	4 (0.11%)	14 (0.13%)		0 (0%)	2 (0.18%)		0 (0%)	1 (0.13%)		0 (0%)	1 (0.21%)	
Current smoking (%)			0.17 ^b^			0.62 ^b^			0.29 ^b^			0.56 ^b^
No	3267 (94.70%)	10,054 (95.28%)		220 (95.24%)	1062 (96.02%)		208 (96.74%)	729 (94.55%)		115 (95.83%)	454 (96.80%)	
Yes	179 (5.19%)	485 (4.60%)		11 (4.76%)	42 (3.80%)		7 (3.26%)	41 (5.32%)		5 (4.17%)	14 (2.99%)	
Missing value	4 (0.11%)	13 (0.12%)		0 (0%)	2 (0.18%)		0 (0%)	1 (0.13%)		0 (0%)	1 (0.21%)	
Betel nut chewing (%)			0.43 ^b^			NA			NA			0.37 ^b^
No	3431 (99.45%)	10,477 (99.29%)		231 (100%)	1099 (99.37%)		214 (99.53%)	767 (99.48%)		119 (99.17%)	466 (99.36%)	
Yes	11 (0.32%)	46 (0.44%)		0 (0%)	4 (0.36%)		0 (0%)	2 (0.26%)		1 (0.83%)	1 (0.21%)	
Missing value	8 (0.23%)	29 (0.27%)		0 (0%)	3 (0.27%)		1 (0.47%)	2 (0.26%)		0 (0%)	2 (0.43%)	
**Comorbidity** ^d^												
Hypertension, yes (%)	76 (2.20%)	385 (3.65%)	**<0.0001** ^b^	65 (28.14%)	333 (30.11%)	0.61 ^b^	31 (14.42%)	186 (24.12%)	**0.003** ^b^	57 (47.50%)	244 (52.03%)	0.43 ^b^
Diabetes, yes (%)	20 (0.58%)	117 (1.11%)	**0.01** ^b^	33 (14.29%)	115 (10.40%)	0.11 ^b^	13 (6.05%)	64 (8.30%)	0.34 ^b^	32 (26.67%)	102 (21.75%)	0.31 ^b^
Hyperuricemia/gout, yes (%)	446 (12.93%)	511 (4.84%)	**<0.0001** ^b^	36 (15.58%)	62 (5.61%)	**<0.0001** ^b^	25 (11.63%)	40 (5.19%)	**0.001** ^b^	41 (34.17%)	52 (11.09%)	**<0.0001** ^b^
Urinary tract stones, yes (%)	9 (0.26%)	21 (0.20%)	0.64 ^b^	2 (0.87%)	5 (0.45%)	0.35 ^b^	1 (0.47%)	5 (0.65%)	1.00 ^b^	0 (0%)	4 (0.85%)	0.59 ^b^
Cancer, yes (%)	628 (18.20%)	2677 (25.37%)	**<0.0001** ^b^	37 (16.02%)	283 (25.59%)	**0.003** ^b^	29 (13.49%)	164 (21.27%)	**0.01** ^b^	39 (32.50%)	172 (36.67%)	0.46 ^b^
**Laboratory examinations**												
Fasting glucose, mg/dL	103.5 ± 24.85	101 ± 19.77	**<0.0001** ^a^	104.1 ± 20.93	99.1 ± 16.32	**0.0002** ^a^	100.9 ± 18.18	99.48 ± 16.77	0.61 ^a^	103.3 ± 23.19	101.4 ± 21.09	0.86 ^a^
Total cholesterol, mg/dL	187.6 ± 34.07	191.1 ± 33.41	**<0.0001** ^a^	196.2 ± 34.53	199.1 ± 32.82	0.24 ^a^	182.5 ± 32.96	179.7 ± 32.04	0.27 ^a^	181.5 ± 34.72	184.1 ± 34.04	0.47 ^a^
Triglyceride, mg/dL	125.4 ± 72.85	110.7 ± 68.77	**<0.0001** ^a^	140.6 ± 67.33	130.2 ± 67.90	**0.003** ^a^	116.5 ± 55.22	98.78 ± 49.62	**<0.0001** ^a^	141.95 ± 72.72	129.8 ± 68.16	0.15 ^a^
HDL, mg/dL	52.8 ± 15.08	56.4 ± 15.26	**<0.0001** ^a^	49.9 ± 13.65	54.3 ± 14.27	**<0.0001** ^a^	54.22 ± 16.23	53.76 ± 14.62	0.88 ^a^	45.13 ± 13.86	50.68 ± 13.41	**<0.0001** ^a^
Uric acid, mg/dL	6.5 ± 1.72	5.5 ± 1.46	**<0.0001** ^a^	6.5 ± 1.80	5.5 ± 1.22	**<0.0001** ^a^	6.475 ± 1.75	5.623 ± 1.34	**<0.0001** ^a^	6.971 ± 1.66	5.671 ± 1.27	**<0.0001** ^a^
BUN, mg/dL	21.5 ± 8.73	15.9 ± 4.33	**<0.0001** ^a^	20.5 ± 6.60	15.77 ± 3.86	**<0.0001** ^a^	20.48 ± 6.50	16.25 ± 4.23	**<0.0001** ^a^	24.65 ± 9.83	15.77 ± 3.97	**<0.0001** ^a^
Creatinine, mg/dL	1.3 ± 0.62	0.9 ± 0.83	**<0.0001** ^a^	1.2 ± 0.32	0.8 ± 0.16	**<0.0001** ^a^	1.288 ± 0.36	0.8855 ± 0.18	**<0.0001** ^a^	1.409 ± 0.55	0.8494 ± 0.17	**<0.0001** ^a^
Triglyceride/HDL, ratio	2.7 ± 2.30	2.2 ± 2.22	**<0.0001** ^a^	3.2 ± 2.28	2.7 ± 2.12	**<0.0001** ^a^	2.4928 ± 1.77	2.1281 ± 2.11	**0.001** ^a^	103.3 ± 23.19	101.4 ± 21.09	**0.003** ^a^
ratio < 3.29	2441 (70.75%)	8746 (82.88%)	**<0.0001** ^b^	144 (62.34%)	828 (74.86%)	**0.002** ^b^	162 (75.35%)	660 (85.60%)	**0.004** ^b^	3.7777 ± 3.18	2.9107 ± 2.10	0.05 ^b^
ratio ≥ 3.29	897 (26.00%)	1802 (17.08%)		80 (34.63%)	278 (25.14%)		48 (22.33%)	111 (14.40%)		70 (58.33%)	323 (68.87%)	
Proteinuria, yes (%)	887 (25.71%)	1322 (12.53%)	**<0.0001** ^b^	73 (31.60%)	176 (15.91%)	**<0.0001** ^b^	72 (33.49%)	132 (17.12%)	**<0.0001** ^b^	49 (40.83%)	146 (31.13%)	**<0.0001** ^b^

Group 1: no dyslipidemia, no CVD; Group 2: dyslipidemia, no CVD; Group 3: no dyslipidemia, CVD; Group 4: dyslipidemia, CVD; Low eGFR was defined as eGFR < 60 mL/min/1.73 m^2^; high eGFR was defined as eGFR ≥ 60 mL/min/1.73 m^2^; Data presented as mean ± standard deviation or number of participants (% of variables); ^a^ *t*-test; ^b^ Chi-Square test. ^c^ Underweight: BMI < 18.5; Normal: 8.5 ≤ BMI < 24; Overweight: 24 ≤ BMI < 27; Obese: BMI ≥ 27; ^d^ Some participants had multiple comorbidities; **Bold** font indicates statistical significance (*p* < 0.05); Abbreviation: eGFR, estimated glomerular filtration rate; BMI, body mass index; HDL, high density lipoprotein; BUN, blood urea nitrogen; *p* value was not applicable due to the zero cell in chi-square test.

**Table 3:** Effect of risk factors on reduced eGFR by univariate logistic regression models.

	Group 1	Group 2	Group 3	Group 4
	OR (95% CI)	OR (95% CI)	OR (95% CI)	OR (95% CI)
**Demographics**				
Age	**1.08 (1.08–1.09)**	**1.08 (1.05–1.11)**	**1.06 (1.04–1.08)**	**1.11 (1.08–1.15)**
Gender				
Male (vs. female)	**1.29 (1.19–1.39)**	**1.37 (1.02–1.83)**	1.06 (0.78–1.44)	1.37 (0.92–2.05)
Marital status				
Married/cohabiting	1	1	1	1
Widowed/divorced/separated	**1.29 (1.19–1.42)**	0.85 (0.57–1.24)	**1.55 (1.09–2.18)**	1.37 (0.84–2.19)
Never married	1.16 (0.96–1.39)	0.38 (0.06–1.30)	1.23 (0.51–2.67)	0.59 (0.03–3.39)
BMI ^a^ level				
Normal weight	1	1	1	1
Underweight	1.09 (0.89–1.31)	0.80 (0.30–1.81)	1.82 (0.87–3.62)	0.93 (0.14–3.69)
Overweight	**1.21 (1.11–1.32)**	1.33 (0.95–1.84)	1.41 (0.99–2.01)	1.40 (0.88–2.23)
Obese	**1.53 (1.37–1.69)**	**1.74 (1.18–2.55)**	**2.22 (1.46–3.36)**	1.36 (0.79–2.29)
**Socioeconomics**				
Educational attainment				
Without high school diploma school (vs. with high school diploma or higher)	**1.34 (1.24–1.44)**	1.32 (0.98–1.78)	0.91 (0.66–1.26)	1.26 (0.79–1.98)
Income level				
Poor (vs. not poor)	**1.87 (1.51–2.32)**	4.82 (0.58–40.35)	1.27 (0.46–3.11)	1.96 (0.09–20.65)
**Lifestyle**				
Exercise habit				
Regular	1	1	1	1
No	1.04 (0.92–1.17)	0.95 (0.58–1.52)	0.98 (0.58–1.61)	1.51 (0.74–2.99)
Occasional	**1.11 (1.01–1.22)**	1.08 (0.74–1.58)	1.21 (0.83–1.78)	**1.98 (1.15–3.46)**
Alcohol drinking (vs. no)	1.05 (0.95–1.16)	0.94 (0.65–1.33)	1.16 (0.80–1.65)	1.42 (0.88–2.26)
Current smoking (vs. no)	1.14 (0.95–1.35)	1.26 (0.61–2.41)	0.59 (0.24–1.27)	1.41 (0.45–3.77)
Betel nut chewing (vs. no)	0.73 (0.36–1.36)	NA	NA	3.92 (0.15–99.51)
**Comorbidity ^b^**				
Hypertension (vs. no)	**0.59 (0.46–0.76)**	0.91 (0.66–1.24)	**0.53 (0.35–0.79)**	0.83 (0.56–1.25)
Diabetes (vs. no)	**0.52 (0.31–0.82)**	1.44 (0.94–2.16)	0.71 (0.37–1.28)	1.31 (0.82–2.06)
Hyperuricemia/gout (vs. no)	**2.92 (2.55–3.33)**	**3.11 (1.99–4.79)**	**2.41 (1.41–4.04)**	**4.16 (2.58–6.69)**
Urinary tract stones (vs. no)	1.31 (0.57–2.78)	1.92 (0.27–8.98)	0.72 (0.04–4.47)	NA
Cancer (vs. no)	**0.66 (0.59–0.72)**	**0.56 (0.38–0.79)**	**0.58 (0.37–0.87)**	0.83 (0.54–1.27)
**Laboratory examinations**				
Fasting glucose, mg/dL	**1.01 (1.00–1.01)**	**1.01 (1.01–1.02)**	1.01 (0.99–1.01)	1.00 (0.99–1.01)
Total cholesterol, mg/dL	**0.99 (0.99–0.99)**	0.99 (0.99–1.00)	1.00 (0.99–1.01)	0.99 (0.99–1.00)
Triglyceride, mg/dL	**1.00 (1.00–1.00)**	**1.00 (1.00–1.00)**	**1.01 (1.00–1.01)**	1.00 (1.00–1.01)
HDL, mg/dL	**0.98 (0.98–0.99)**	**0.98 (0.97–0.99)**	1.00 (0.99–1.01)	**0.97 (0.95–0.98)**
Uric acid, mg/dL	**1.54 (1.49–1.59)**	**1.67 (1.48–1.89)**	**1.47 (1.31–1.66)**	**1.88 (1.56–2.30)**
BUN, mg/dL	**1.19 (1.19–1.21)**	**1.21 (1.17–1.26)**	**1.18 (1.14–1.23)**	**1.25 (1.19–1.33)**
Triglyceride/HDL, ratio	**1.11 (1.09–1.13)**	**1.09 (1.03–1.16)**	**1.08 (1.01–1.17)**	**1.14 (1.05–1.23)**
ratio ≥ 3.29 (vs. ratio <3.29)	**1.78 (1.63–1.96)**	**1.66 (1.22–2.24)**	**1.76 (1.19–2.56)**	**1.55 (1.02–2.34)**
Proteinuria (vs. no)	**2.43 (2.21–2.67)**	**2.44 (1.77–3.36)**	**2.43 (1.73–3.41)**	**2.94 (1.93–4.49)**

Group 1: no dyslipidemia, no CVD; Group 2: dyslipidemia, no CVD; Group 3: no dyslipidemia, CVD; Group 4: dyslipidemia, CVD; **Bold** font indicates statistical significance (*p* < 0.05); ^a^ Underweight: BMI < 18.5; Normal: 8.5 ≤ BMI < 24; Overweight: 24 ≤ BMI < 27; Obese: BMI ≥ 27; ^b^ Some participants had multiple comorbidities; Abbreviation: eGFR, estimated glomerular filtration rate; CI, confidence interval; OR, odds ration; Ref, reference; BMI, body mass index; HDL, high density lipoprotein; BUN, blood urea nitrogen; NA, not applicable due to the zero cell.

**Table 4:** Effect of risk factors on reduced eGFR by multivariate logistic regression models.

	Group 1	Group 2	Group 3	Group 4
	aOR (95% CI)	aOR (95% CI)	aOR (95% CI)	aOR (95% CI)
**Demographics**				
Age	**1.06 (1.05–1.07)**	**1.06 (1.03–1.10)**	1.03 (0.99–1.06)	1.04 (0.98–1.11)
Gender				
Male (vs. female)	**0.60 (0.53–0.68)**	0.65 (0.41–1.01)		
Marital status				
Married/cohabiting	1		1	
Widowed/divorced/separated	1.07 (0.93–1.21)		**1.80 (1.15–2.82)**	
Never married	1.04 (0.79–1.35)		2.06 (0.79–4.96)	
BMI ^a^ level				
Normal weight	1	1	1	
Underweight	0.95 (0.71–1.25)	1.01 (0.24–3.32)	1.43 (0.55–3.45)	
Overweight	1.10 (0.97–1.25)	1.08 (0.69–1.69)	1.09 (0.69–1.69)	
Obese	1.13 (0.97–1.31)	0.81 (0.46–1.39)	1.26 (0.72–2.16)	
**Socioeconomics**				
Educational attainment				
Without high school diploma school (vs. with high school diploma or higher)	**1.16 (1.03–1.29)**			
Income level				
Poor (vs. not poor)	1.12(0.82–1.51)			
Lifestyle				
Exercise habit				
Regular	1			1
No	1.09 (0.94–1.27)			0.72 (0.22–2.18)
Occasional	1.09 (0.97–1.22)			**2.39 (1.05–5.69)**
**Comorbidity ^b^**				
Hypertension (vs. no)	**0.44 (0.29–0.66)**		0.59 (0.32–1.04)	
Diabetes (vs. no)	0.92 (0.39–1.94)			
Hyperuricemia/gout (vs. no)	**1.94 (1.60–2.35)**	**2.06 (1.07–3.89)**	**3.37 (1.57–7.27)**	0.88 (0.32–2.22)
Cancer (vs. no)	0.90 (0.79–1.03)	0.63 (0.38–1.03)	0.65(0.36–1.12)	
**Laboratory examinations**				
Fasting glucose, mg/dL	0.99 (0.99–1.01)	1.01 (0.99–1.02)		
Total cholesterol, mg/dL	**0.99 (0.99–1.00)**			
Triglyceride, mg/dL	**1.00 (1.00–1.00)**	1.00 (0.99–1.01)	**1.01 (1.00–1.02)**	
HDL, mg/dL	1.00 (0.99–1.01)	0.99 (0.96–1.01)		0.99 (0.95–1.02)
Uric acid, mg/dL	**1.43 (1.38–1.49)**	**1.29 (1.11–1.51)**	**1.21 (1.06–1.38)**	**1.57 (1.19–2.12)**
BUN, mg/dL	**1.18 (1.17–1.19)**	**1.17 (1.12–1.22)**	**1.15 (1.10–1.21)**	**1.27 (1.18–1.38)**
Triglyceride/HDL, ratio				
ratio ≥ 3.29 (vs. ratio < 3.29)	**1.31 (1.09–1.55)**	0.87 (0.45–1.66)	0.77 (0.37–1.55)	0.83 (0.29–2.27)
Proteinuria (vs. no)	**1.70 (1.48–1.95)**	**1.78 (1.11–2.81)**	1.32 (0.83–2.08)	2.10 (0.98–4.51)

Group 1: no dyslipidemia, no CVD; Group 2: dyslipidemia, no CVD; Group 3: no dyslipidemia, CVD; Group 4: dyslipidemia, CVD; **Bold** font indicates statistical significance (*p* < 0.05); ^a^ Underweight: BMI < 18.5; Normal: 8.5 ≤ BMI < 24; Overweight: 24 ≤ BMI < 27; Obese: BMI ≥ 27; ^b^ Some participants had multiple comorbidities; Abbreviation: eGFR, estimated glomerular filtration rate; CI, confidence interval; aOR, adjusted odds ration; Ref, reference; BMI, body mass index; HDL, high density lipoprotein; BUN, blood urea nitrogen.
